# Photodegradation of a Broad-Spectrum Antibiotic Azithromycin Using H_2_O_2_ under Ultraviolet Irradiation

**DOI:** 10.3390/ijms25126702

**Published:** 2024-06-18

**Authors:** Nasser Ibrahim Zouli

**Affiliations:** Department of Chemical Engineering, College of Engineering, Jazan University, Jazan 45142, Saudi Arabia; nizouli@jazanu.edu.sa

**Keywords:** azithromycin, UV irradiation, photodegradation, HPLC determination, H_2_O_2_, zero-order reaction kinetics

## Abstract

The photodegradation of azithromycin present was carried out in water using H_2_O_2_ under UV irradiation. The reaction variables considered in this study were the amount of H_2_O_2_ solution and the initial concentration of azithromycin to evaluate the performance of the photodegradation process. The azithromycin degradation was not observed in the dark during stirring for 20 min. The study showed an efficient photodegradation of azithromycin using H_2_O_2_ as an oxidant in the presence of UV irradiation. The azithromycin degradation was altered significantly by the pH of the irradiated solution. The degradation was low at an acidic pH and showed an increasing trend as the pH changed to basic. The azithromycin degradation increased with a higher amount (higher concentration) of H_2_O_2_. The degradation of azithromycin decreased with a higher concentration of azithromycin in the reacting solution. The highest degradation of AZT was achieved in 1 h using a 1.0 ppm AZT solution containing 3 mL of H_2_O_2_. The experimental data obtained were well-fitted to zero-order reaction kinetics. The results of this study were found quite excellent. They showed 100% degradation in 1 h when compared with those reported in the literature, both with photocatalysis using nanomaterials and photolysis using light irradiation and/or H_2_O_2_. The UV/H_2_O_2_ system was found to be quite efficient for the photodegradation of azithromycin, and this system can be applied to degrade other organic pollutants present in industrial wastewater.

## 1. Introduction

Researchers all over the world are trying to eliminate organic pollutants from wastewater using several techniques such as adsorption, photolysis, photocatalysis, electrochemical conversion, etc. These organic pollutants consist of dyes, pharmaceuticals, surfactants, etc. Organic dyes are degraded in water by photocatalysis using different types of nanocomposites. For example, photocatalysts have been used to eliminate methylene [1,2], methyl orange and Eriochrome black T [3], reactive red 250 dye [4], methylene blue and methyl orange [5], rhodamine B and crystal violet [6], methyl orange and alizarin red [7], and Congo red and methylene blue [8]. Similarly, pharmaceuticals present in wastewater have been eliminated by photocatalysis. Hussain et al. [9] used a photo-Fenton process involving MnO_2_ and moving bed biofilm treatment followed by ozonation and achieving more than 90% removal for Ibuprofen and Ofloxacin. Likewise, photocatalysis has been used for the degradation and elimination of azithromycin (AZT) antibiotic that has been used successfully and widely in the treatment of COVID-19, as well as against several other infections. AZT belongs to a class of medications called macrolide antibiotics. Because of its wide use in the treatment of coronavirus conditions as well as in other infectious diseases, its concentration in wastewater has been quite significantly increased in recent years and thus poses health hazards for humans and other living organisms. One of the effective ways to remove AZT from the environment is photodegradation either through photolysis or photocatalytic degradation. Several investigators have used a variety of semiconductor nanomaterials to eliminate AZT and other related drugs. Over the years, several types of nanomaterial-based catalysts have been synthesized and used under variable conditions of pH, catalyst doping, and AZT concentration. These nanomaterials contain titanium, iron, zinc, molybdenum, cobalt, and other metals mostly in oxide form alone or with other metals. Other research studies have been conducted using only UV or visible irradiation (photolysis), and have employed H_2_O_2_ as an oxidant. Some of these studies are reported here. Sharma et al. [10] used Cu_2_O–TiO_2_ nanotubes for the photodegradation of AZT and achieved complete degradation within 90 min in a solution having 100 μg/mL AZT concentration and 1.5 g/L of nanotubes at a pH of 7 under visible light irradiation. Ling et al. [11] synthesized a g-C_3_N_4_/Fe-MCM-48 nano-size composite with a 3D structure, and used it for the photocatalytic ozonation of AZT under simulated solar light. The degradation achieved was almost 100% within 11 min. Shajahan and AbuHaija [12] used CuFe_2_O_4_, binary CdS/CuFe_2_O_4,_, and ternary g-C_3_N_4_/CdS/CuFe_2_O_4_ nanocomposite for the photodegradation of AZT using a visible light source. The ternary nanocomposite exhibits the highest degradation efficiency of 85% within 90 min. Mehrdoost et al. [13] utilized PAC/Fe/Ag/Zn nanocomposites for photodegradation by UV irradiation and achieved 99.5% degradation of AZT (10 mg/L) at a pH of  9 within 120 min irradiation time using 0.04 g/L of PAC/Fe/Ag/Zn. Mohammed et al. [14] used ZnO/Co_3_O_4_ nanocomposites and reported 71.7% degradation of AZT within 80 min under visible light irradiation. Shukla et al. [15] reported a 40% degradation of AZT (10 mg/L) using ZnO nanoparticles under UV irradiation. Kumar et al. [16] reported 98.4% AZT photodegradation in 90 min under visible light using a Ag@Bi_4_O_5_I_2_/SPION@calcium alginate catalyst. Ospino-Atehortúa et al. [17] used persulfate and simulated sunshine achieving about 70% of AZT photodegradation in 120 min of irradiation in basic pH. Tenzin et al. [18] utilized SrTiO_3_ for the photodegradation of AZT under UV irradiation. The photodegradation at a pH of 2 was 55% in 150 min of irradiation, while at a pH of 12, it was 98% in 90 min. Li et al. [19] used a MoS2-GO composite for the photocatalytic degradation of AZT (100 mg/L) under visible light and reported 87% degradation within 3 h. Rueda-Márquez et al. [20] used natural solar irradiation with immobilized TiO_2_ and achieved more than 80% degradation of AZT in urban wastewater effluents. Sayadi et al. [21] used 1.0 g/L of GO@Fe_3_O_4_/ZnO/SnO_2_ nanocomposites and achieved 90% of AZT (initial AZT 30 mg/L) within 120 min of UV-C irradiation at a pH of 3. Čizmic et al. [22] investigated the use of nanostructured TiO_2_ film (comprised of pure anatase) and achieved AZT degradation using UV-C irradiation at a pH of 10. Naraginti et al. [23] used a nanorod ternary nanocomposite ZrO_2_/Ag@TiO_2_ and attained 90% of AZT (initial AZT 20 mg/L) in 480 min of visible light irradiation. Biancullo et al. [24] investigated TiO_2_-based photocatalytic treatment using UVA-LEDs for wastewater samples spiked with AZT and reported complete AZT degradation within 60 min using 1 g/L of TiO_2_. Luo et al. [25] used La-TiO_2_/AC nanocomposites for the photocatalytic degradation of AZT under UV irradiation in wastewater and reported that at a pH of 4 and AZT concentration of 10 mg/L, irradiation for 90 min degraded 95.6% of AZT.

Some investigators have reported the use of photolysis using UV or visible irradiation with or without oxidants. Chen [26] performed the photolysis of AZT under visible and UV light irradiation and concluded that UV irradiation provided higher degradation (82.6%) than visible light (53.1%) using an AZT concentration of 10 mg/L at a pH of 4. Cano et al. [27] used simulated sunlight radiation with H_2_O_2_ and accomplished full AZT degradation in 120 min of photolysis. Voigt et al. [28] achieved the photoinduced degradation of AZT using UVC/VUV irradiation. Tong et al. [29] reported the photolysis of AZT under simulated solar radiation.

The literature cited above has shown a variety of nanomaterials used for AZT elimination from water and the effect of H_2_O_2_ on the photodegradation of AZT, as well as studies utilizing photolysis without the use of nanomaterials. In this study, we utilized UV irradiation with H_2_O_2_ to perform AZT degradation without using any solid expensive catalytic nanomaterials. When H_2_O_2_ is exposed to light irradiation, the photolysis of hydrogen peroxide occurs and produces hydroxyl radicals (•OH) which are highly reactive. The highly reactive hydroxyl radicals created during this process oxidize and break down a variety of organic contaminants. The idea of using H2O2 as an oxidant and not using any expensive catalysts was also aimed at economizing the research study and to achieve the research goals with minimum chemicals, materials, effort, and time. These features of the study were very important during the scaling up of this photodegradation process.

**Figure 1:**
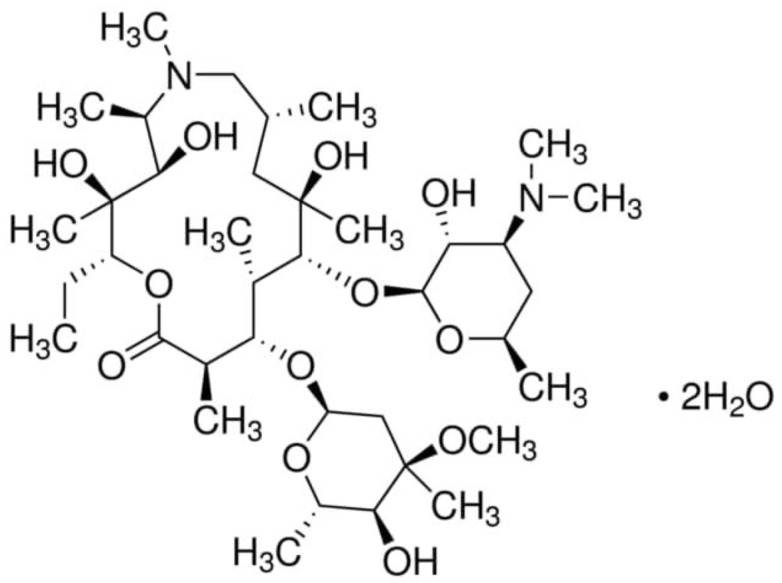
The molecular structure of AZT (molecular formula: C_38_H_72_N_2_O_12_, molecular weight: 749.0 g/mol).

The parameters studied were the pH of the irradiated solution and variable concentrations of H_2_O_2_ and AZT in the solution. First, the pH of the solution was varied to determine the effect of pH on the degradation efficiency of AZT. Then, keeping the optimized pH constant (at which maximum AZT degradation was achieved), the concentration of H_2_O_2_ was varied followed by varying the concentration of AZT. Figure 1 shows the molecular structure of AZT. The AZT molecule has a 15-membered lactone ring, multiple hydroxyl (OH) groups, amino sugars attached to the macrolide ring, and contains various methyl (CH3) and methoxy (OCH3) groups. The IUPAC name for AZT is as follows: (2R,3S,4S,5R,8R,10R,11R,12S,13S,14R)-2-ethyl-3,4,10-trihydroxy-3,5,6,8,10,12,14-heptamethyl-11-[[3,4,6-trideoxy-4-(dimethylamino)-3-(2,4-dimethoxyphenyl)-2-(methylamino)-β-D-lyxo-hexopyranosyl]oxy]-1-oxa-6-azacyclopentadecan-15-one.

## 2. Results and Discussion

### 2.1. Effect of Solution pH on the AZT Degradation Efficiency

**Figure 2:**
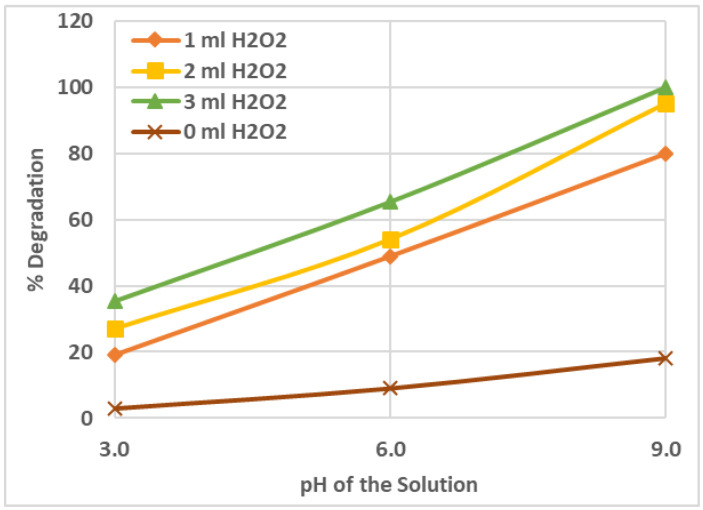
Percent degradation of AZT as a function of pH of the solution at different H_2_O_2_ concentrations. Experimental conditions: 50 mL solution of AZT 1.0 ppm concentration, 60 min irradiation time, UV irradiation intensity 50 mW/cm^2^, 30% H_2_O_2_ solution addition of 0, 1, 2, and 3 mL.

Figure 2 shows the result of varying the pH of the irradiated solution on the percent photodegradation of AZT. The degradation was low, at a pH of 3 (acidic). The degradation was increased upon increasing the pH to 6 and it was further increased to a pH of 9 (basic). The complete degradation of AZT was achieved at a pH of 9 using a 50 mL solution having an AZT concentration of 1.0 ppm, with an addition of 3 mL of 30% H_2_O_2_ solution, which was irradiated with UV light for 60 min. At a higher pH in the basic region, the degradation of AZT increased due to the higher concentration of OH radicals produced which attacked the AZT molecules more frequently and converted them into degraded products and finally into CO_2_ and H_2_O [27].

### 2.2. Effect of Amount/Concentration of H_2_O_2_ in the Reacting Solution

**Figure 3:**
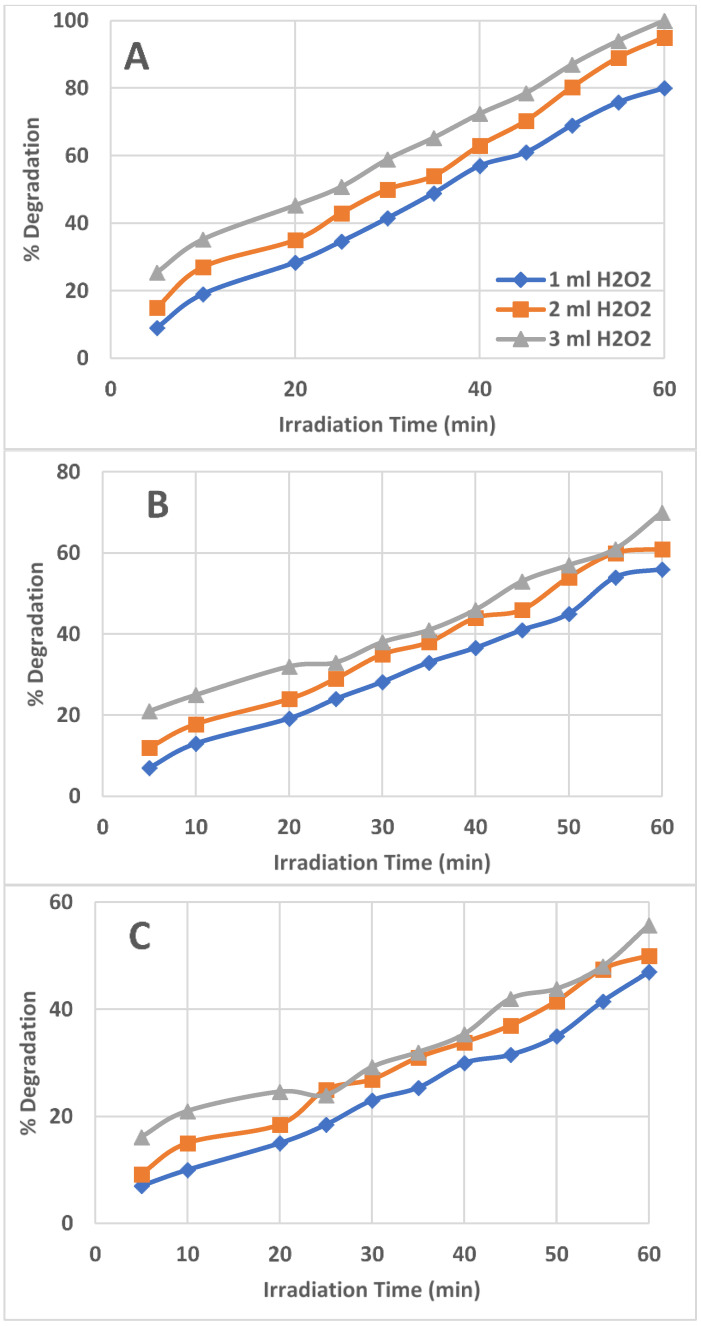
Percent degradation of AZT with respect to the initial AZT concentration and variable amount of H_2_O_2_ in 50 mL of irradiated solution, (**A**) = 1.0 ppm AZT solution, (**B**) = 3.0 ppm AZT solution, (**C**) = 5.0 ppm AZT solution. Irradiated AZT solution volume 50 mL, pH 9, and UV irradiation intensity 50 mW/cm^2^.

The effect of the amount of 30% H_2_O_2_ (1, 2, and 3 mL) in the photocatalytic degradation of AZT was recorded. It was witnessed that the AZT degradation was increased with increasing amounts of H_2_O_2_ solution and with increasing UV irradiation time. Figure 3 shows the percent degradation of AZT for variable amounts of H_2_O_2_ using three different initial AZT concentrations (1, 3, and 5 ppm). The reason was that a higher amount of H_2_O_2_ provided a higher amount of OH radicals generated due to the interaction with UV light irradiation. As a result, more and more AZT molecules were attacked by highly reactive OH radicals and experienced breakdown; thus, the percentage of AZT photodegradation was higher.

### 2.3. Effect of Initial AZT Concentration on AZT Degradation

The initial AZT concentration varied from 1 to 3 and 5 ppm to observe the effect on the percent degradation of AZT. As shown in Figure 3, at a low AZT concentration of 1 ppm (Figure 3A), the highest and complete degradation of AZT was observed. Figure 3A shows that the 3 mL H_2_O_2_ solution added provided the highest and complete (100%) degradation of AZT in a solution having 1 ppm AZT, in 60 min of UV irradiation, followed by 2 mL H_2_O_2_ solution, which provided 95% AZT degradation. The 1 mL H_2_O_2_ yielded 80% AZT degradation. The AZT percent degradation was low at a 3 ppm initial AZT concentration which was further reduced in the case of 5 ppm AZT concentration (Figure 3B and 3C). The full breakdown of AZT was promoted greatly with a greater concentration of 30% H_2_O_2_ solution. Reaction solutions containing 1 and 3 ppm of AZT showed a greater rate of AZT degradation than those containing 5 ppm of AZT. Using 3 mL of a 30% H_2_O_2_ solution, the maximum and highest deterioration trend was noted for the 1 ppm AZT solution.

### 2.4. Kinetics of AZT Photodegradation

To decide on the kinetics of the AZT photodegradation reaction, three plots were developed which are as follows: (1). Ct/Co versus t (if the plot is linear, then it is a zero-order kinetic reaction), rate = k; (2). Ln Ct/Co versus t (if linear, then it is a first-order kinetic reaction), rate = k[Ct]; (3). 1/Ct /Co versus t (if linear, then it is a second-order kinetic reaction), rate = k[Ct]^2^.

**Figure 4:**
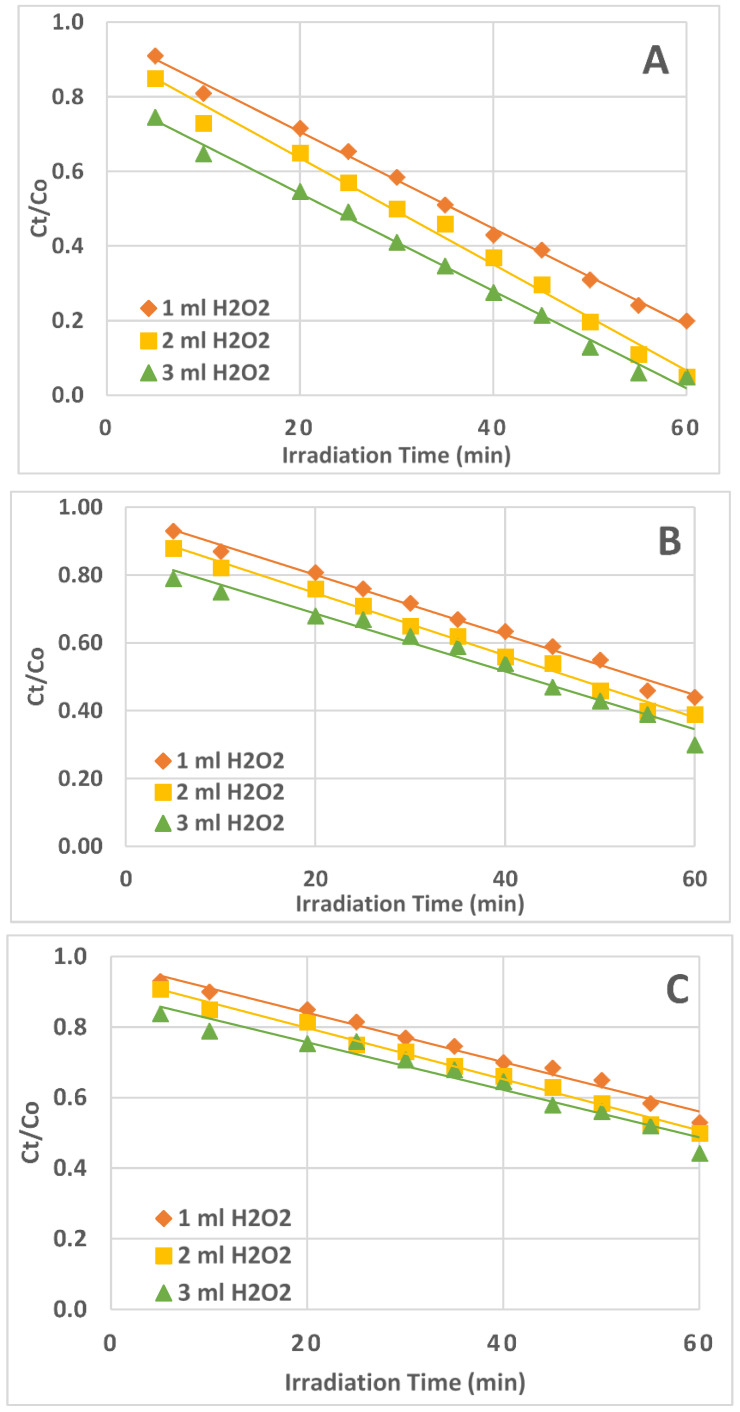
Plots of Ct/Co vs. irradiation time (t) for the zero-order reaction kinetics. (**A**) = 1.0 ppm AZT solution, (**B**) = 3.0 ppm AZT solution, (**C**) = 5.0 ppm AZT solution. Irradiated solution volume 50 mL, pH 9, and UV irradiation intensity 50 mW/cm^2^.

**Figure 5:**
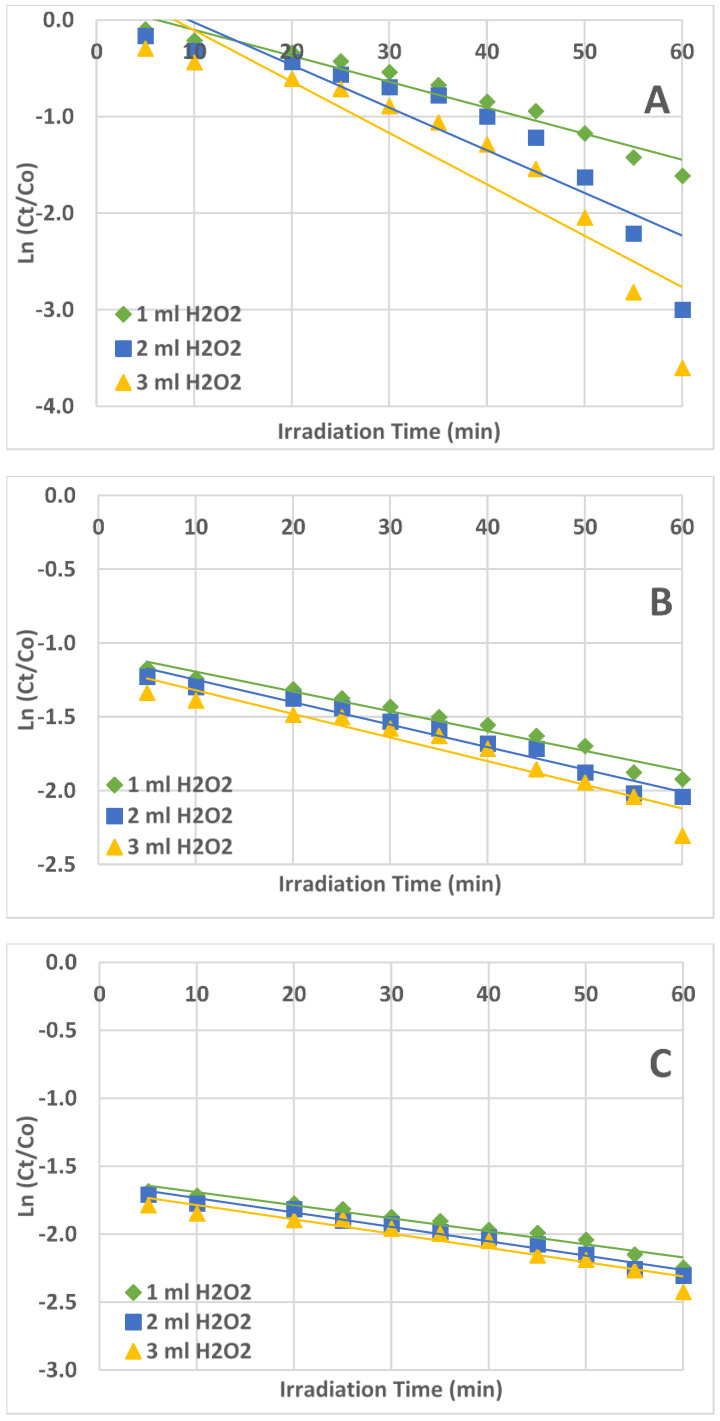
Plots of Ln (Ct/Co) vs. irradiation time (t) for the first-order reaction kinetics. (**A**) = 1.0 ppm AZT solution, (**B**) = 3.0 ppm AZT solution, (**C**) = 5.0 ppm AZT solution. Irradiated solution volume 50 mL, pH 9, and UV irradiation intensity 50 mW/cm^2^.

**Figure 6:**
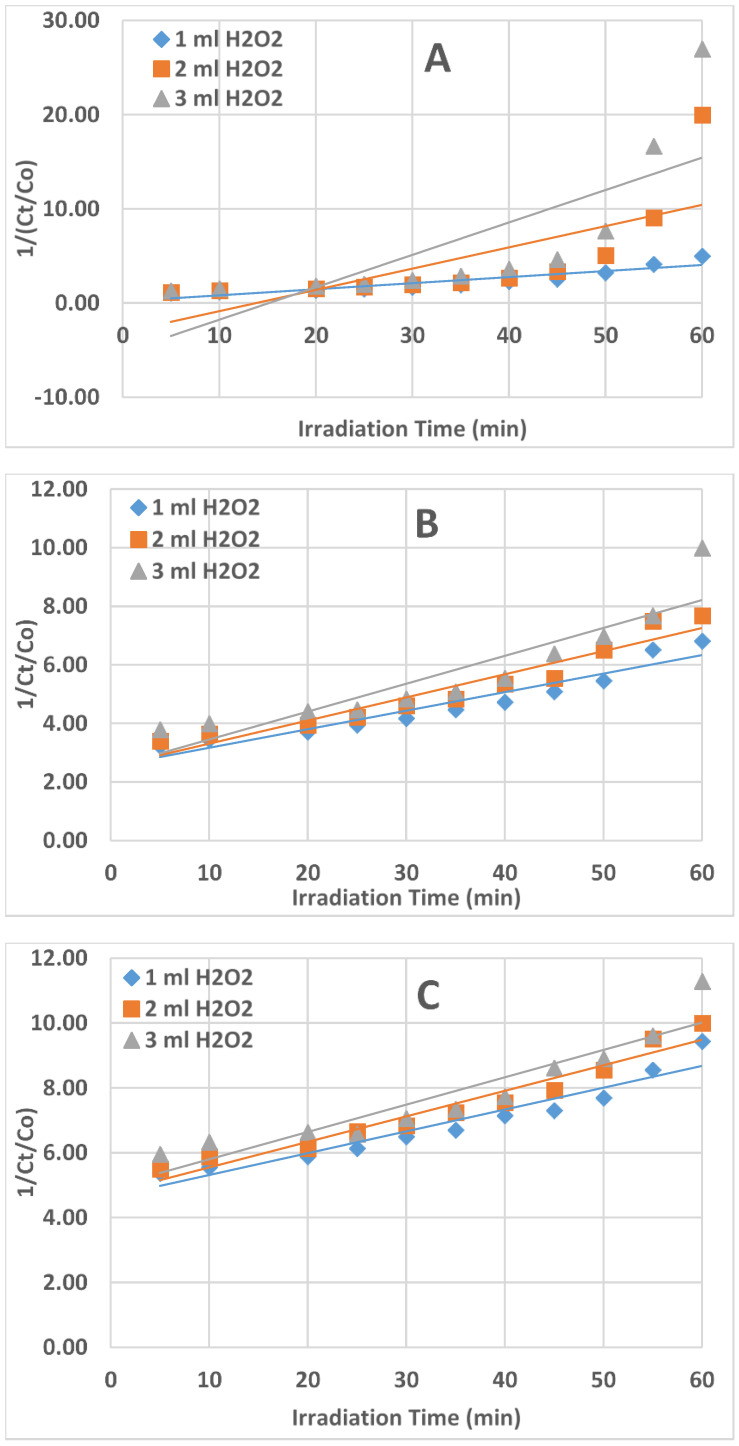
Plots of 1/(Ct/Co) vs. irradiation time (t) for the second-order reaction kinetics. (**A**) = 1.0 ppm AZT solution, (**B**) = 3.0 ppm AZT solution, (**C**) = 5.0 ppm AZT solution. Irradiated AZT solution volume 50 mL, pH 9, and UV irradiation intensity 50 mW/cm^2^.

The plot of Ct/Co vs. t (Figure 4) produced the best straight-line equation among the three plots (Figure 4, Figure 5 and Figure 6); the slope of the equation represented the rate constant (k). This confirmed that the UV/H_2_O_2_ reaction system’s AZT photodegradation reaction followed zero-order kinetics.

The values of the rate constant (k) and correlation coefficient (R^2^) for the degradation of AZT under UV irradiation for the zero, first, and second orders of reaction are shown in Table 1. The obtained correlation coefficient values are near unity, suggesting a good fit between the data and the zero-order kinetics. The correlation coefficient values obtained for first and second-order kinetics are quite far from unity.

### 2.5. Photodegradation Mechanism

The photodegradation of AZT with H_2_O_2_ under UV irradiation is believed to proceed by the following reactions:

H_2_O_2_ + hν → OH^●^ (1)

2H_2_O_2_ → 2H_2_O + O_2_ (2)

e^−^ + H_2_O_2_ → OH^●^ + OH^−^ (3)

h^+^ + OH^−^ → OH^●^ (4)

e^−^ + O_2_ → O_2_^●−^ (5)

OH^●^ + AZT → Intermediates → CO_2_ + H_2_O (6)

O_2_^●−^ + AZT → Intermediates → CO_2_ + H_2_O (7)

**Figure 7:**
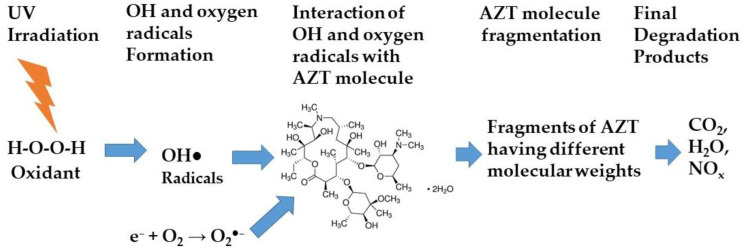
Scheme of AZT photodegradation using H_2_O_2_ under UV irradiation.

First, the H_2_O_2_ under UV irradiation produced OH radicals (OH^●^), which are quite reactive species, and attacked the AZT molecules. This resulted in the fragmentation of AZT molecules into different species that are further oxidized and converted into intermediates and then finally into CO_2_, H_2_O, and NO_x_. According to this chemical pathway, the key step is the generation of hydroxyl radicals from the photolysis of H_2_O_2_ which then reacts with the AZT molecules. In addition, the O_2_^●−^ radicals generated also attack the AZT molecules and degrade them into intermediates and finally into CO_2_, H_2_O, and NO_x_. The scheme of AZT photodegradation using H_2_O_2_ under UV irradiation is illustrated in Figure 7.

### 2.6. Comparison of This Work with the Literature

Table 2 presents a comparison of the photodegradation efficiency of this research work with the literature-reported results. The comparison is made with those results produced with and without catalysts. The results of this study were quite excellent and showed 100% degradation in 1 h when compared with those reported in the literature, both with photocatalysis using nanomaterials and photolysis using light irradiation and/or H_2_O_2_.

## 3. Materials and Methods

### 3.1. Materials

The chemicals used in this study were 98.0% azithromycin dehydrate (molecular formula C_38_H_72_N_2_O_12_●2H_2_O and molecular weight 785.0) and 30% hydrogen peroxide (H_2_O_2_) solution, purchased from Sigma Aldrich (Burlington, MA, USA). NaOH and HCl were obtained from Alfa-Aesar (Ward Hill, MA, USA) and were used to maintain the pH of the irradiated solution. All chemicals used were of analytical grade. All the AZT-containing solutions were prepared in drinking water.

### 3.2. Methods

**Figure 8:**
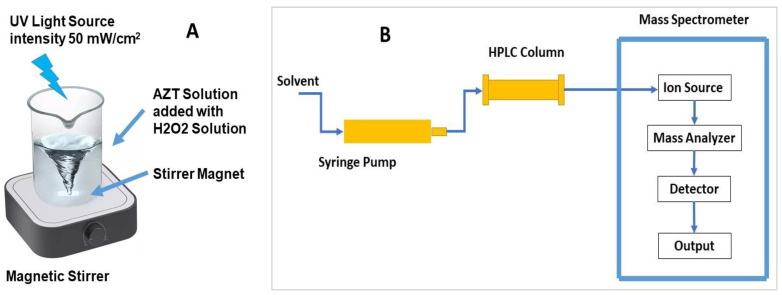
(**A**) An aqueous solution containing AZT and H_2_O_2_ on a magnetic stirrer cum hot plate irradiated with UV light having 50 mW/cm^2^ intensity, input voltage 230 V, and 50/60 Hz. (**B**) Schematic diagram of the HPLC-MS analyzer.

The AZT solutions of 1.0, 3.0, and 5.0 ppm concentration were prepared in bottled drinking water. Of the AZT solution, 50 mL from each concentration was taken in separate glass beakers and a known amount of 30.0% H_2_O_2_ solution was added. The mixture was agitated for 20 min in the dark to check for AZT degradation with H_2_O_2_ in the dark. Then, the mixture was irradiated with UV light under continuous stirring. Figure 8A shows an aqueous solution containing AZT and H_2_O_2_ on a magnetic stirrer cum hot plate, which was irradiated with UV light having 50 mW/cm^2^ intensity, 230 V input voltage, and 50/60 Hz. The external temperature of the sample was maintained at 40 °C during irradiation. After every 5 min, 1.0 mL of the solution was sampled from the beaker and analyzed for AZT concentration by the HPLC system. There was no need to separate the solid catalyst from the sampled solution using a centrifuge since no solid catalyst was used in the degradation process. The pH of the solution was adjusted using dilute solutions of NaOH and HCl. The separation of the components was accomplished on a reverse-phase C_18_ column (5.0 μm, 250.0 mm × 4.6 mm) installed in an HPLC analytical system (Waters Corporation (Milford, MA, USA)) coupled with a triple–quadrupole mass spectrometer. A schematic diagram of the HPLC-MS analyzer is shown in Figure 8B. The mobile phase was optimized to achieve a fast separation of the components of the solution at a flow rate of 1.5 mL/min. An isocratic methanol/buffer mobile phase at the ratio of 90:10 *v*/*v* provided the separation of the components [35]. The components of the solution were eluted from the column, injected into the mass spectrometer, and converted into mass spectra, which were matched with the MS library for identification. The mass spectrometer was operated in multiple-reaction monitoring mode, with a capillary voltage of 3.9 kV, cone voltage of 50 V, and multiplier voltage of 650 V. Nitrogen was supplied to nebulizing, desolvation, and cone gas, with a collision gradient of 1.0 and UHP argon as the collision gas. Using this method, all irradiated solutions were HPLC-chromatographed and the amounts of unconverted AZT present in the solutions were determined. The concentration of AZT present in the irradiated solutions was the unconverted AZT amount that provided the percent degradation of AZT. The parameters studied were the pH of the solution (3, 6, and 9), the concentration of H_2_O_2_ (1, 2, and 3 mL), and the concentration of AZT (1, 3, and 5 ppm). For determining the effect of pH, the AZT concentration was 1 ppm, irradiation time 60 min, UV irradiation intensity 50 mW/cm^2^, and the amounts of H_2_O_2_ added were 1, 2, and 3 mL. Once the pH of the solution was optimized, it was kept and the concentration of H_2_O_2_ was varied followed by varying the concentration of AZT.

## 4. Conclusions

This study shows the efficient UV irradiation-based photodegradation of AZT using H_2_O_2_ as an oxidant. The effects of variable concentrations of AZT and H_2_O_2_ present in the irradiated solution have been demonstrated successfully. In the dark, with stirring, the degradation of AZT was not observed in the presence of H_2_O_2_. The AZT degradation efficiency was affected drastically by the pH of the irradiated solution. The AZT degradation rate was low at an acidic pH and showed an increasing trend as the pH changed to basic. The highest AZT degradation was observed at a pH of 9. The percent degradation of AZT decreased with a higher initial AZT concentration. The degradation of AZT was found to increase with a higher amount (higher concentration) of H_2_O_2_. The obtained experimental data confirmed that the photodegradation of AZT with H_2_O_2_ under UV irradiation followed zero-order reaction kinetics, as it fits very well with the plots of Ct/Co vs. t. It is predicted that the OH radicals (OH●) and oxygen radicals (O_2_●^−^) generated during the breakdown of H_2_O_2_ attack AZT molecules and produce AZT radicals (AZT●). These AZT radicals were converted to intermediate fractions and finally transformed into CO_2_ and H_2_O. The results of this study were quite excellent. They showed 100% degradation in 1 h when compared with those reported in the literature, both with photocatalysis using nanomaterials and photolysis using light irradiation and/or H_2_O_2_. Without the need for a solid catalyst, the photodegradation of AZT by the UV/H_2_O_2_ system was found to be highly effective. This system can also be used to degrade other organic contaminants common in industrial effluents. Future research on the removal of AZT from actual wastewater containing other organic contaminants ought to be carried out with continuously stirred reactors, as well as using UV irradiation with different wavelengths and intensities. Other oxidants and initiators can also be used to see their impact on the degradation of AZT and other organic pollutants present in wastewater.

## Figures and Tables

**Table 1:** Values of the rate constant (k) and correlation coefficient (R^2^) of the first, second, and zero reaction orders obtained for AZT degradation under UV light irradiation.

	Zero-Order Kinetics (Ct/Co) vs. t		First-Order Kinetics Ln (Ct/Co) vs. t		Second-Order Kinetics 1/(Ct/Co) vs. t	
1 ppm AZT solution (50 mL)	R^2^	k, min^−1^	R^2^	k, min^−1^	R^2^	k, min^−1^
1 mL 30% H_2_O_2_	0.9968	0.0130	0.9555	0.0269	0.8403	0.0643
2 mL 30% H_2_O_2_	0.9912	0.0142	0.8293	0.0442	0.5231	0.2255
3 mL 30% H_2_O_2_	0.9974	0.0134	0.8387	0.0532	0.5816	0.3440
3 ppm AZT solution (50 mL)	R^2^	k, min^−1^	R^2^	k, min^−1^	R^2^	k, min^−1^
1 mL 30% H_2_O_2_	0.9925	0.0089	0.9670	0.0134	0.9188	0.0633
2 mL 30% H_2_O_2_	0.9923	0.0092	0.9697	0.0152	0.9229	0.0792
3 mL 30% H_2_O_2_	0.9760	0.0085	0.9207	0.0160	0.8311	0.0952
5 ppm AZT solution (50 mL)	R^2^	k, min^−1^	R^2^	k, min^−1^	R^2^	k, min^−1^
1 mL 30% H_2_O_2_	0.9848	0.0070	0.9599	0.0325	0.9208	0.0673
2 mL 30% H_2_O_2_	0.9908	0.0073	0.9755	0.0281	0.9451	0.0787
3 mL 30% H_2_O_2_	0.9576	0.0067	0.9220	0.0210	0.8717	0.0844

R^2^ = correlation coefficient, k = rate constant.

**Table 2:** The photodegradation efficiency of this study compared with the literature-reported results.

No.	Catalyst	Process Conditions	Degradation Efficiency	Reference
1.	10% Cu_2_O/TiO_2_ nanotubes, 1.5 g/L	Visible light irradiation, AZT 100 μg/mL, pH 7	100% in 1.5 h	[11]
2.	PAC/Fe/Ag/Zn, 0.04 g/L	UV irradiation, pH 9, AZT 10 mg/L, pseudo-first-order kinetic	99% in 2 h	[13]
3.	Ag@Bi_4_O_5_I_2_/SPION@calcium alginate	Visible light (300 W Xe lamp), AZT 10 mg/L, 0.3 mg/mL	98% in 1.5 h	[16]
4.	K_2_S_2_O_8_ 5.0 to 80.0 mg/L	Simulated solar irradiation 30 min (1.5 KW xenon lamp, 290–800 nm), 50.0 mL solution, AZT 1.0 mg/L, pH 5	70% in 2 h	[17]
5.	SrTiO_3_, 30 mg	UV irradiation, AZT 20 mg/L, pH 12, pseudo-first-order reaction kinetics	99% in 4 h	[18]
6.	(a) MoS_2_ (b) MoS_2_-GO	Visible light irradiation, AZT 100 mg/L	(a) 75% in 3 h (b) 87% in 3 h	[19]
7.	TiO_2_ P25, 227 ng/L (UV), 250 ng/L (solar)	UV irradiation 55 Wm^−2^, Solar 33 W m^−2^, domestic wastewater	52% (UV), 87% (solar)	[20]
8.	GO@Fe_3_O_4_/ZnO/SnO_2,_ 1 g/L,	UV-C Irradiation, AZT 30 mg/L, pH 3	90% in 2 h	[21]
9.	ZrO_2_/Ag@TiO_2_	Visible light irradiation, AZT 20 mg/L (50 mL solution)	90% in 8 h	[23]
10.	La-TiO_2_/active carbon	UV irradiation, pH 4, AZT 10 ppm	96% in 1.5 h	[25]
11.	Fe (III)-oxalate	UV irradiation, AZT 10 mg/L, pH 4,	83% in 2 h	[26]
12.	35% H_2_O_2_, 482.0 ppm as oxidant	Simulated sunlight irradiation 500 W/m^2^, AZT 1 ppm (50 mL solution), pH 9	100% in 2 h	[27]
13.	No catalyst, only UV irradiation,	Xenon arc lamp (power 500 watts, UV power: 765 W/m^2^, Wavelength 300–800 nm), AZT 1.0 μg/L, pH 7, Temp. 40 °C	90% in 7 days	[30]
14.	7.5 mg/L FeSO_4_ + 27.5 mg/L H_2_O_2_	Simulated solar irradiation, UV power 50 mW/cm^2^, Wavelength 290–800 nm, AZT 3 mg/L, pH 3	92% in 0.5 h	[31]
15.	Gd3+ doped BiVO_4_, catalyst 2 g/L	UV-LED irradiation, Wavelength 370 nm, power 4.65 mW/cm^2^, domestic wastewater	63% in 3 h	[32]
16.	No catalyst, only UV irradiation,	UV irradiation power 163 mW/cm^2^, AZT 110 mg/L, pH 7. Temp. 65 °C	73% in 0.8 h	[33]
17.	Photo-Fenton catalyst, Fe^2+^, 20 mg/L, H_2_O_2_ 50 mg/L	Solar UV power 2.65 mW/cm^2^, Reactor volume 2 × 15 L, AZT 25 ng/L, pH 7	24% in 3 h	[34]
18.	3 mL of 30% H_2_O_2_ solution	UV irradiation intensity 50 mW/cm^2^, AZT 1.0 ppm (50 mL solution), pH 9.0, zero-order reaction kinetics	100% in 1 h	This work

## Data Availability

The data presented in this study are available on request from the corresponding author due to privacy restrictions.
